# Survey on the prevalence of intestinal parasites in domestic cats (*Felis catus* Linnaeus, 1758) in central Nepal

**DOI:** 10.1002/vms3.999

**Published:** 2022-11-08

**Authors:** Roshan Babu Adhikari, Madhuri Adhikari Dhakal, Purna Bahadur Ale, Ganga Ram Regmi, Tirth Raj Ghimire

**Affiliations:** ^1^ Third Pole Conservancy Bhaktapur Nepal; ^2^ Nepalese Army Institute of Health Sciences (NAIHS) Kathmandu Nepal; ^3^ Department of Microbiology Dorevitch Pathology Albury New South Wales Australia; ^4^ Department of Zoology Tri‐Chandra Multiple Campus Tribhuvan University Kathmandu Nepal

**Keywords:** *Cystoisospora*, feral cats, polyparasitism, *Toxoascaris*, zoonosis

## Abstract

**Introduction:**

Cats (*Felis catus*) are the only felines that live in close contact with humans. Since cats can act as vectors, carriers, reservoirs and definitive hosts of many gastrointestinal (GI) parasites, parasitic assessment could contribute to their survival and well‐being.

**Aims:**

The current study aimed to assess the diversity and prevalence of GI parasites in domestic and feral cats from Ratnanagar in Chitwan in Central Nepal.

**Methods:**

A total of 107 fresh faecal samples of cats (90 household cats and 17 feral cats) of varied ages and sex were collected and transported to the laboratory. The copromicroscopic examination was carried out following direct wet mount, formalin‐ethyl acetate sedimentation, saturated salt flotation, acid‐fast staining and sporulation techniques. Furthermore, associated risk factors were evaluated to ascertain the predictor of risks for parasitic acquisition.

**Results:**

The current study revealed an overall 95.3% prevalence rate with a 100% rate in feral cats and 94.4% in household cats. Altogether, 18 (17 known and one unknown) different species of GI parasites were reported with the helminths (95.3%; 11 species) and the protozoa (55.1%; seven species). Besides age and sex, outdoor lifestyle, absence or unknown history of medication and hunting behaviour of the felines are the predictors of risk. Furthermore, mixed infection was comparatively higher than single infection in the faecal samples.

**Conclusions:**

Cats harbour a higher prevalence and greater diversity of GI parasites, and parasitism varies with age and sex. This finding can be essential for veterinarians and public health authorities for strategic treatment and for assessing the zoonotic transmission of the parasites from these felines. Importantly, an effective medication strategy for cats and owners is recommended.

## Graphical abstract

The current study determined the prevalence and diversity of gastrointestinal parasites in domestic and feral cats from Ratnanagar in Chitwan in Central Nepal. It revealed a 100% rate in feral cats and 94.4% in household cats.

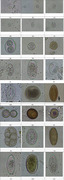

## 1 INTRODUCTION

Domestic cats (*Felis catus* Linnaeus, 1758) with Taxonomic Serial Number 183798 (www.itis.gov) are the only felines living exclusively in close contact with humans. Ecologically, they are the world's most invasive species (Medina et al., 2011) and can be found in almost every terrestrial habitat (Trouwborst et al., 2020). Because of their ability to hunt rodents and for companionship, these mammals are mostly domesticated as pets (Hill, 2008). However, they are reared as a mouser in agricultural regions in rural areas.

While the cats control the rodent population and increase the crop yields and safety of the stored grains, disease caused by gastrointestinal (GI) parasites leads to high morbidity (Yang & Liang, 2015) and mortality (Parsons, 1987). These parasites are protozoa like *Blastocystis*, *Cryptosporidium*, *Entamoeba*, *Giardia*, *Cystoisospora* and *Toxoplasma* and helminths like acanthocephalans, *Capillaria, Dicrocoelium, Dipylidium*, hookworms, *Joyexiella*, *Physaloptera*, *Strongyloides*, *Toxocara*, *Troglostrongylus* and tapeworms (Khalafalla, 2011; Mircean et al., 2010; Nyambura Njuguna et al., 2017; Traversa & Di Cesare, 2016; Yang & Liang, 2015). These pathogens cause anaemia, anorexia, broncho‐pulmonary infection, coughing, dehydration, dermatitis, diarrhoea, hepatomegaly, mucous or bloody faeces, ocular lesions, pale mucous membranes, potbellied appearance, systemic lymph adenomegaly, vomiting, weight loss and others in these hosts (Brianti et al., 2012; Crisi et al., 2018; Irwin & Traub, 2006; Nguyen et al., 2017; Parsons, 1987; Traversa & Di Cesare, 2016). Furthermore, a cat heavily infected with GI parasites becomes more susceptible to secondary infection caused by a virus, bacterium, or fungus (Barrs et al., 1999; Diakou et al., 2021). In addition, cats act as the definitive host, reservoir and carrier of a few potentially serious and zoonotically important GI parasites like *Toxoplasma gondii* (Torda, 2001), *Toxocara cati* (Borecka & Klapec, 2015; Cong et al., 2014), hookworm (*Ancylostoma ceylanicum;* Yang & Liang, 2015), *Paragonimus* (Liu et al., 2008), and *Echinococcus multiloculari*s (Thompson, 2015) and cause clinical diseases. In this context, free‐ranging pet cats and feral cats contribute to the spillover of microorganisms to the nearby wildlife and humans (Adhikari et al., 2020; Dickman, 1996; Trouwborst et al., 2020). Interestingly, parasite spillover impacts predation, fear, competition and hybridisation of the sympatric wildlife (Trouwborst et al., 2020).

In addition, the presence of infected cats around human inhabitants may pose a severe public health concern. However, the prevalence and diversity of GI parasites in cats in Nepal have not been fully explored yet. Therefore, the objectives of the current study were to determine the diversity and prevalence of GI parasites among domestic and feral cats and to analyse how these parasites could affect feline health. These findings will be important for treating parasites in cats and controlling possible zoonosis.

## 2 MATERIALS AND METHODS

### 2.1 Study area

**FIGURE 1:**
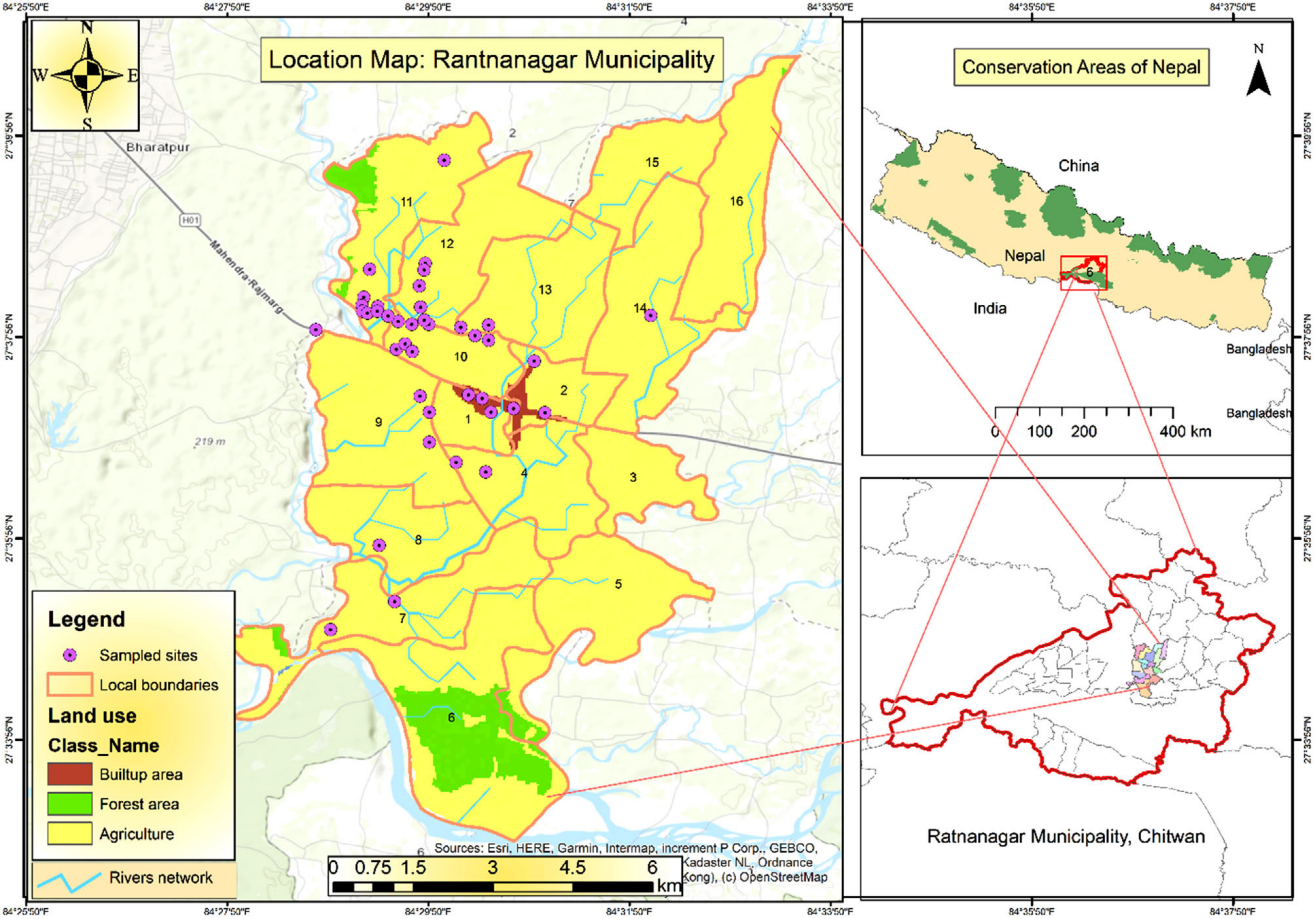
Map of study area

The current study was conducted in Ratnanagar Municipality in eastern Chitwan in Central Nepal (Figure 1). It is 151 km away from the capital city (Kathmandu). Its climate is tropical to subtropical. The average annual maximum temperature is 21–35°C and the average minimum temperature is 10–24°C with an average annual precipitation range (5–530 mm; https://www.worldmeteo.info/en/asia/nepal/ratnanagar/weather‐244941/). Most people depend on agriculture like animal husbandry, pisciculture, poultry, apiculture, cropping and horticulture. They domesticate cats as pets mostly without proper care and with easy outdoor access. The pets usually live in and around the human inhabitant (Supporting Information 1) and are fed milk, rice and meat. The pets are set free to hunt rodents that destroy the stored grains. In contrast, people never domesticate feral cats, but these animals live freely nearby human settlements. These cats mainly consume rodents, fish, frogs, birds and small to larger insects, especially by hunting.

### 2.2 Sample collection, preservation and transportation

The faecal sampling had been conducted from 2 May to 30 November 2020. For household cats, we employed the door‐to‐door visit method. For convenience, the owners/farmers were instructed to keep their cats indoors overnight and the collection was performed the next morning. The details on age, sex, medication and rearing practices adopted were noted. Furthermore, opportunistic selection of the faecal samples was employed for feral cats. Since feral cats are pretty shy in nature, they avoid crowded environments and are likely to bury their faeces. Thus, they were followed quietly until they defecate. Furthermore, artificial sand trays/boxes were also placed in different core sites. By nature, feral cats are highly suspicious to any things new, so we further kept some foods like bread in and around such trays to attract them to it and waited until they accept these trays and defecate over there. The stool samples were also collected from the gardens, edges of roads and agricultural fields. Finally, 107 fresh faecal samples (*n* = 107) were collected that included 90 faeces from household cats (*n*1 = 90) and 17 faeces from feral cats (*n*2 = 17). Their population was classified into two groups: young: ≤ 12 months of age and old/adult cats: > 12 months based on the direct observation of the fresh faecal pellets. The collected faecal samples were carefully observed for their consistency and the presence of blood, mucus, adult nematodes and detached segments of cestodes in the sampling sites and then simultaneously preserved in 2.5% weight/volume (w/v) potassium dichromate solution in 20 ml sterile vials. The samples were then transported to Animal Research Laboratory, Faculty of Science, Nepal Academy of Science and Technology, Lalitpur, Nepal, for additional investigation and microscopic observation.

### 2.3 Laboratory processing and examination

The copromicroscopic examination of the faecal samples was carried out with five different routine laboratory techniques like direct wet mount, formalin‐ethyl acetate sedimentation, saturated sodium chloride salt flotation (45% w/v NaCl), acid‐fast staining and sporulation assays as previously explained (Adhikari & Ghimire, 2021; Adhikari et al., 2020; Adhikari et al., 2021; Adhikari et al., 2021; Adhikari  et al., 2021; Adhikari et al., 2022; Aryal et al., 2021; Ghimire & Bhattarai, 2019; Ghimire et al., 2021).

For direct wet mount, simply a drop of well‐mixed faecal sample in 2.5% potassium dichromate was kept on the glass slide and then observed under the microscope.

For formalin‐ethyl acetate sedimentation, 2 gm of the faecal sample was mixed with normal saline (0.85% NaCl) in a 15 ml centrifuge tube. It was then centrifuged at 1200 rpm for 5 min and the supernatant was discarded. The sediment was further added with 10 ml 10% formalin and 4 ml ethyl acetate and re‐centrifuged (1200 rpm X 5 min) for better separation of debris. Finally, the supernatant layers were discarded, and a single drop of the sediment was taken for microscopic observation.

For the flotation technique, the sediment obtained after the first centrifugation was added with 12 ml of 45% w/v NaCl (floating media) and re‐centrifuged (1200 rpm X 5 min). Further, the floating media was added drop by drop until the tube was completely filled and a cover slip was placed at its mouth, such that the coverslip touched the floating media. After 10 min, the coverslip was carefully removed for microscopic observation.

Finally, for acid‐fast staining, the sediment after formalin‐ethyl acetate sedimentation was conducted for thin smear preparation. The smears were dried at room temperature and other procedure follows the chronological order (a) fixation in absolute methanol for 2 min, (b) counter‐staining with carbol fuchsin for 15 min that follows destaining with acid alcohol and (c) restaining with malachite green for a minute that followed a gentle wash with distilled water. Finally, the smears were dried and observed under the microscope with immersion oil.

For sporulation, 1:4 ratios of coccidian parasite‐positive faecal sample and potassium dichromate were incubated at 28°C, respectively. In every 24 h interval, the sporulation state of the coccidian was examined via the flotation technique.

### 2.4 Parasite identification

All the faecal samples were observed under a compound microscope (Optika Microscopes Italy, B‐383PLi) at a total magnification of 100X, 400X and 1000X. The camera (SXView 2.2.0.172 Beta, 6 November 2014, copyright (C) 2013–2014) was used to capture the images of the parasites. Identification of the parasites was carried out based on size assessed by ImageJ 1.51k (National Institute of Health and literature previously described (Dubey, 1976; Lucio‐Forster et al., 2012; Soulsby, 2012; Zajac et al., 2021). Since the oocysts of both these coccidia (*T. gondii* and *Hammondi hammondi*) are very difficult to detect morphologically as well as serologically (Yang & Liang, 2015), we named them *T. gondii/H. hammondi*.

### 2.5 Data analysis

All the data generated were expressed as numbers of positive samples and prevalence rates in the table using Microsoft Word 2010. Further, GraphPad Software (Prism 5 for Windows Version 5.00 @ 1992–2007 GraphPad Software, Inc.) was used to analyse the data. The chi‐square test was used to evaluate *p‐*values that compare the different available variables. A *p*‐value less than 0.05 (*p* < 0.05; 95% confidence interval) was considered for statistical significance.

## 3 RESULTS

The macroscopic observation was carried out in all the faecal samples (*n* = 107) that had been collected from the sampling sites. Five different types of faecal samples were recorded. The study of faecal consistency found 46 formed stool (43%), 33 mixed stool (30.8%), 14 half‐formed and half diarrhoeal (13.1%), 19 mainly formed and few softer stool (17.8%), 16 mushy and lumpy stool (15%), seven hard‐constipated stool (6.5%) and five diarrhoeal stool (4.7%). Except for five stool samples of formed consistency, all rest stool types were positive for parasites (Supporting Informations 2 and 3).

In the current study, the total prevalence of GI parasites was 95.3%, with 94.4% (85/90) in household cats and 100% (17/17) in feral cats. Importantly, 16 different species (protozoa: six and helminths: 10) were reported in household cats and 16 different species (protozoa: seven and helminths: nine) were reported in feral cats aggregating a total of 18 different species (protozoa: seven and helminths: 11) among the overall population (Supporting Information 4; Table 1). The overall prevalence of helminths (92.5%) was significantly higher than that of protozoa (55.1%). Interestingly, three morphotypes of strongyle eggs (size range: 68–80 × 32–51 µm), two morphotypes of *Strongyloides* egg (size range: 50–60 × 34–37), and four morphotypes of oocysts of *Sarcocystis* spp. (size range: 16–23 × 11–17 µm) were reported. The prevalence of each 17 parasitic species was compared with that reported in different countries (Table 2).

**TABLE 1:** Parasites detected in the faecal samples of household and feral cats (*N* = 107). Young: ≤ 1 year, adult: >1 year

	Young (≤ 1 year)			Adult (>1 year)			
Gastrointestinal (GI) parasites	Male (20)	Female (15)	Total (*n* = 35)	Male (25)	Female (47)	Total (n = 72)	Overall prevalence rate (*N* = 107)
**Sarcodina**							
*Entamoeba* sp.	2 (10%)	3 (20%)	5 (14.3%)	6 (24%)	5 (10.6%)	11 (16.7%)	16 (15%)
**Coccidia**							
*Cystoisospora rivolta*	2 (10%)	1 (6.7%)	3 (8.6%)	6 (24%)	10 (21.3%)	16 (22.2%)	19 (17.8%)
*Cystoisospora felis*	3 (15%)	2 (13.3%)	5 (14.3%)	5 (20%)	9 (19.1%)	14 (19.4%)	19 (17.8%)
*Sarcocystis* spp.	4 (20%)	0 (0%)	4 (11.4%)	4 (16%)	9 (19.1%)	13 (18.1%)	17 (15.9%)
*Toxoplasma*/ *Hammondia*	3 (15%)	1 (6.7%)	4 (11.4%)	2 (8%)	7 (14.9%)	9 (12.5%)	13 (12.1%)
*Cryptosporidium* sp.	3 (15%)	3 (20%)	6 (17.1%)	4 (16%)	2 (4.3%)	6 (8.3%)	12 (11.2%)
Unknown coccidia	0 (0%)	0 (0%)	0 (0%)	0 (0%)	1 (2.1%)	1 (1.4%)	1 (0.9%)
**Total protozoa**	**9 (45%)**	**7 (46.7%)**	**16 (45.7%)**	**15 (60%)**	**28 (80%)**	**43 (59.7%)**	**59 (55.1%)**
**Nematoda**							
*Ancylostoma tubaeforme*	12 (60%)	7 (46.7%)	19 (54.3%)	15 (60%)	31 (66%)	46 (63.9%)	65 (60.7%)
*Toxocara cati*	11 (55%)	9 (60%)	20 (57.1%)	7 (28%)	16 (34%)	23 (31.9%)	43 (40.2%)
*Ancylostoma braziliense*	5 (25%)	2 (13.3%)	7 (20%)	7 (28%)	13 (27.7%)	20 (27.8%)	27 (25.2%)
*Capillaria* sp.	0 (0%)	0 (0%)	0 (0%)	5 (20%)	9 (19.1%)	14 (19.4%)	14 (13.1%)
Strongyle	1 (5%)	0 (0%)	1 (3.1%)	5 (20%)	5 (10.6%)	10 (13.9%)	11 (10.3%)
*Strongyloides* sp.	0 (0%)	1 (6.7%)	1 (2.9%)	2 (8%)	4 (8.5%)	6 (8.3%)	7 (6.5%)
*Toxoascaris leonina*	0 (0%)	0 (0%)	0 (0%)	3 (12%)	0 (0%)	3 (4.2%)	3 (2.8%)
**Cestoda**							
*Dipylidium caninum*	0 (0%)	0 (0%)	0 (0%)	2 (8%)	1 (2.1%)	3 (4.2%)	3 (2.8%)
*Hymenolepis* sp.	0 (0%)	0 (0%)	0 (0%)	1 (4%)	1 (2.1%)	2 (2.8%)	2 (1.9%)
Taeniid	0 (0%)	1 (6.7%)	1 (3.1%)	10 (40%)	12 (25.5%)	22 (30.6%)	23 (21.5%)
**Acanthocephala**							
Archiacanthocephala	0 (0%)	0 (0%)	0 (0%)	1 (4%)	1 (2.1%)	2 (2.8%)	2 (1.9%)
**Total Helminths**	**17 (85%)**	**12 (80%)**	**29 (82.9%)**	**25 (100%)**	**45 (95.7%)**	**70 (93.3%)**	**99 (92.5%)**
**Overall Prevalence**	**19 (95%)**	**12 (80%)**	**31 (88.6%)**	**25 (100%)**	**46 (97.9%)**	**71 (98.6%)**	**102 (95.3%)**
**Concurrency**							
Single	4 (20%)	3 (20%)	7 (20%)	0 (0%)	1 (2.1%)	1 (1.4%)	8 (7.5%)
Double	5 (25%)	1 (6.7%)	6 (17.1%)	6 (24%)	11 (23.4%)	17 (23.6%)	23 (21.5%)
Triple	8 (40%)	8 (53.3%)	16 (45.7%)	10 (40%)	24 (51.1%)	34 (47.2%)	50 (46.7%)
Quadruple	2 (10%)	0 (0%)	2 (5.7%)	3 (12%)	8 (17%)	11 (15.3%)	13 (12.1%)
Pentuple	0 (0%)	0 (0%)	0 (0%)	5 (20%)	2 (4.3%)	7 (9.7%)	7 (6.5%)
Hexuple	0 (0%)	0 (0%)	0 (0%)	1 (4%)	0 (0%)	1 (1.4%)	1 (0.9%)

*Note*: Male vs. female: *p* = ns. Helminths in male vs. female: *p* < 0.05. Protozoans in male vs. female: *p* = ns.

**TABLE 2:** Comparison of individual parasitic prevalence of current study with findings from different countries

**Parasites**	**Current prevalence**	**Previous prevalence**	**Country reported**	**Citations**
*Cystoisospora felis*	17.80%	4.80% 27.50%	Bangladesh Indonesia	Mehedi et al. (2020) Rabbani et al. (2020)
*Cystoisospora rivolta*	17.80%	13.20% 11.39% 8.90%	Florida, US China Italy	Wyrosdick et al. (2017) Yang and Liang (2015) Mircean et al. (2010)
*Sarcocystis* spp.	15.90%	17.60% 0.56% 1% 1.30%	Egypt China Italy US	Elsheikha et al. (2006) Yang and Liang (2015) Mircean et al. (2010) Wyrosdick et al. (2017)
*Cryptosporidium* sp.	11.20%	40.80% 10% 3.80% 6.60% 6.20%	Kenya Australia US US China	Nyambura Njuguna et al. (2017) McGlade et al. (2003) Wyrosdick et al. (2017) Spain et al. (2001) Li et al. (2019)
*Toxoplasma gondii/H. hammondi*	12.10%	88% 8.50% 4.65% 2.90%	India Malaysia Iraq Kenya	Borkataki et al. (2013) Ngui et al. (2014) Al‐Aredhi (2015) Nyambura Njuguna et al. (2017)
*Entamoeba* sp.	15%	12% 4.65% 2.90%	Malaysia Iraq Kenya	Ngui et al. (2014) Al‐Aredhi (2015) Nyambura Njuguna et al. (2017)
*Ancylostoma tubaeforme*	60.70%	91% 47.50% 9.60% 6.55%	Spain US Brazil Bangladesh	Millán and Casanova (2009) Liotta et al. (2012) Coelho et al. (2011) Mehedi et al. (2020)
*Ancylostoma braziliensis*	25.20%	50.70% 67.30% 41.60%	Brazil Brazil US	Coelho et al. (2011) Ramos et al. (2013) Liotta et al. (2012)
*Toxocara cati*	40.20%	65.30% 63% 48% 40% 33% 35% 17.70%	Brazil South Korea Malaysia Indonesia US Spain Bangladesh	Monteiro et al. (2016) Lee et al. (2019) Ngui et al. (2014) Rabbani et al. (2020) Spain et al. (2001) Millán and Casanova (2009) Mehedi et al. (2020)
*Toxocara leonina*	2.80%	63% 10.33% 8%	South Korea Indonesia Malaysia	Lee et al. (2019) Rabbani et al. (2020) Ngui et al. (2014)
*Capillaria* sp.	13.10%	14.80% 3.10% 5.50% 3.40%	Europe Italy Italy Brazil	Giannelli et al. (2017) Mircean et al. (2010) Traversa et al. (2009) Ramos et al. (2013)
Strongyle	10.30%	3.8%‐ 78.10% 50.00% 5.60%	Europe Europe Italy	Giannelli et al. (2017) Knaus et al. (2011) Mircean et al. (2010)
*Strongyloides* sp.	5.60%	65.31% 33.50% 28% 43.70% 0.70%	Brazil Australia India Kenya Thailand	Monteiro et al. (2016) Speare and Tinsley (1987) Borkataki et al. (2013) Nyambura Njuguna *et al.* (2017) Rojekittikhun et al. (2014)
Taeniid	21.50%	22% 40% 3.10% 2.70%	Spain India Bangladesh Italy	Millán and Casanova (2009) Borkataki et al. (2013) Mehedi et al. (2020) Mircean et al. (2010)
*Dipylidium caninum*	2.80%	65.31% 8.70% 0.20%	Brazil Kenya Italy	Monteiro et al. (2016) Nyambura Njuguna et al. (2017) Mircean et al. (2010)
*Hymenolepis* sp.	1.90%	1%	Malaysia	Ngui et al. (2014)
Archiacanthocephala	1.90%	1.90% 3.42%	Kenya Brazil	Nyambura Njuguna et al. (2017) Ramos et al. (2013)

Considering the age, adult cats had a higher prevalence rate (98.6%) of enteric parasites than young cats (88.6%); however, the data were not significant (*p* > 0.05). The adults also harboured a more diverse parasite species than the youngs (18 vs. 12 species). Interestingly, *Cryptosporidium* and *Toxocara* were dominant in the young cats, whereas ancylostomatids and taeniids were dominant in the adults. Uniquely, *Capillaria*, *Dipylidium, Toxoascaris, Hymenolepis*, Archiacanthocephala and an unknown coccidian were found in the adults (Table 1; Figure 2). Interestingly, regarding the rate of total parasites as well as protozoa, male cats had a higher rate than females; however, the data were not significant (*p* > 0.05). Further, males had a significantly higher prevalence of helminth species, compared to females (*p* < 0.05). The rate was also higher in young males, compared with young females (95% vs. 80%) or in adult males, compared with adult females (100% vs. 97.9%; Table 1). Besides age and sex differences, other possible predictors of risks like lifestyle, medication and feeding behaviour of the cats were also analysed. Parasitic prevalence was significantly higher in those populations that exclusively live outdoors, have unknown or no history of medication and those that feed exclusively by hunting (Table 3).

FIGURE 2Gastrointestinal parasites identified in the cats. (a) cyst of *Entamoeba* sp. (10 × 10 µm), 400X after direct wet mount technique; (b) oocysts of *Toxoplasma gondii/Hammondi* (11 × 10 µm), (12 × 12 µm); (c) oocyst of *Sarcocystis* sp. 1 (18 × 13 µm), 400X after flotation technique; (d) oocyst of *Sarcocystis* sp. 2 (16 × 11 µm), 400X after flotation technique; (e) oocyst of *Sarcocystis* sp. 3 (21 × 12 µm), 400X after flotation technique; (f) oocyst of *Sarcocystis* sp. 4 (23 × 17 µm), 400X after flotation technique; (g) oocyst of *Cystoisospora felis* (45 × 33 µm), 400X after flotation technique; (h) oocyst of *Cystoisospora rivolta* (25 × 24 µm), 400X after flotation technique; (i) oocyst of unknown coccidia (30 × 29 µm), 400X after flotation technique; (j) egg of *Ancylostoma tubaeforme* (54 × 35 µm), 400X after flotation technique; (k) egg *A. braziliensis* (67 × 37 µm), 400X after flotation technique; (l) egg of *Strongyloides* sp.1. (50 × 37 µm), 400X after flotation technique; (m) egg of *Strongyloides* sp. 2. (60 × 34 µm), 400X after flotation technique; (n) egg of *Toxocara cati* (73 × 65 µm), 400X after formalin‐ethyl acetate sedimentation technique; (o) egg of Archiacanthocephala (86 × 57 µm), 400X after formalin‐ethyl acetate sedimentation technique; (p) eggs of taeniid 1. (28 × 27 and 25 × 24) µm, 400X after formalin‐ethyl acetate sedimentation; (q) egg of taeniid 2. (39 × 36 µm), 400X after formalin‐ethyl acetate sedimentation; (r) egg of taeniid 3. (37 × 31 µm) after formalin‐ethyl acetate sedimentation technique; (s) egg of *Dipylidium caninum* (125 × 79 µm), 400X after formalin‐ethyl acetate sedimentation technique; (t) egg of *Hymenolepis* sp. (52 × 37 µm), 400X after flotation technique; (u) egg of *Capillaria* sp. (58 × 26 µm), 400X after formalin‐ethyl acetate sedimentation technique; (v) egg of strongyle 1. (80 × 36 µm), 400X after formalin‐ethyl acetate sedimentation technique; (w) egg of strongyle 2. (80 × 51 µm), 400X after formalin‐ethyl acetate sedimentation technique; (x) egg of strongyle 3. (68 × 32 µm), 400X after formalin‐ethyl acetate sedimentation technique

**Figure:**
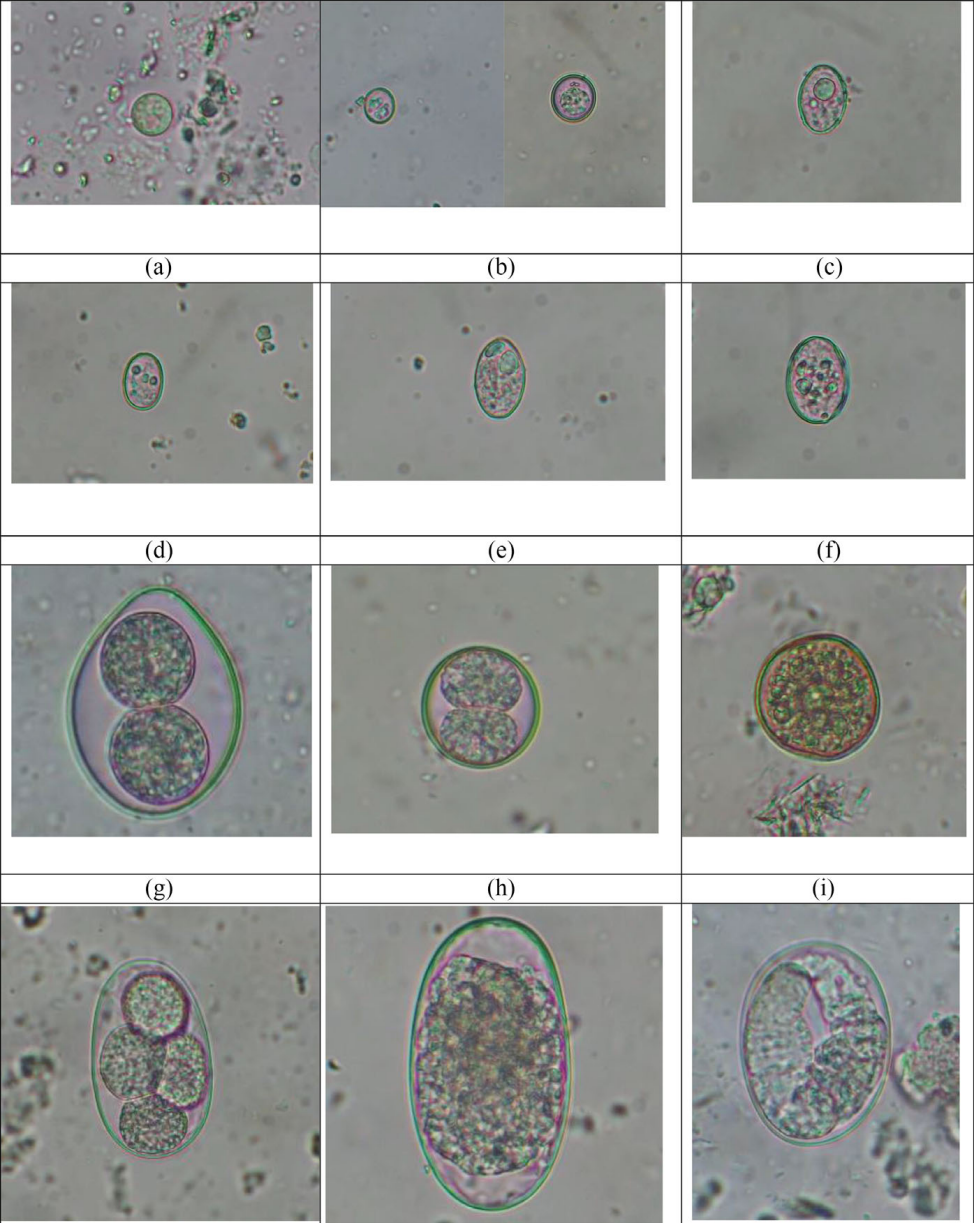

**Figure:**
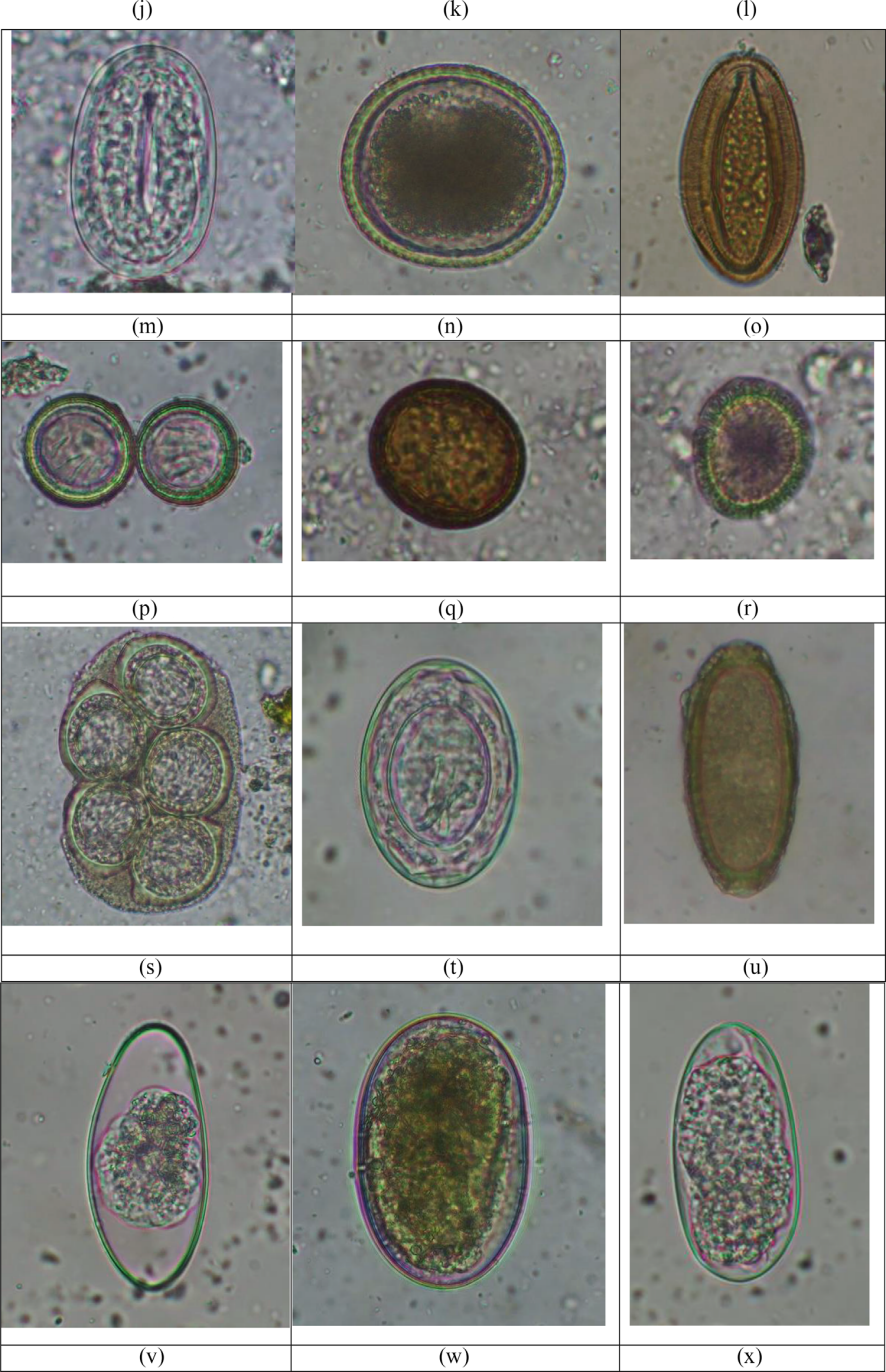

**TABLE 3:** Assessment of risk factors and predictors of risk

**Risk factors**	**Predictors of risk**	**No. of cats**	**Prevalence (%)**	***p*‐values (chi‐square test)**
Lifestyle (rearing practices)	Completely indoor	3	1 (33%)	*p* < 0.05
	Predominantly indoor	12	10 (83%)	
	Predominantly outdoor	75	74 (98.7%)	
	Completely outdoor	17	17 (100%)	
Medication/antiparasitic treatment	6 months	8	4 (50%)	*p* < 0.05
	1 year	15	14 (93.3%)	
	No treatment/unknown history	84	84 (100%)	
Feeding practice	Mother‐dependent	9	7 (77.8%)	*p* < 0.05
	Human feed only	15	12 (80%)	
	Human feed + hunting	66	66 (100%)	
	Exclusively hunting	17	17 (100%)	

Concurrency analysis showed that most cats possessed multiple parasite infections, compared to single infections (87.9% vs. 7.5%). The young cats contained up to four species, whereas the adult cats contained up to six species of parasites (Table 1).

The presence of parasites with respect to specific faecal consistency was also analysed. *Cystoisospora* spp., *Cryptosporidium* sp., and *A. tubaeforme* were dominant in diarrhoeal stools. In contrast, *T. cati* and *A. tubaeforme* were usual in all types of stools (Supporting Information 5).

## 4 DISCUSSION

As per our knowledge, this is the first peer‐reviewed international report regarding the comparative diversity and prevalence of GI parasites in Nepal's domestic and feral cats. The prevalence rate (95.3%; *n* = 107) of GI parasites in domestic cats is similar to that of Iran (94%–95.6%; *n* = 50─113; Arbabi & Hooshyar, 2009; Hajipour et al., 2016), lower than reported that of India (100%; *n* = 100; Borkataki et al., 2013), but higher than those reported from Bangladesh (93.3%; *n* = 30; Barua et al., 2020), United Arab Emirates (87%; *n* = 240; Schuster et al., 2009), Qatar (80%; *n* = 488; Abu‐Madi et al., 2008), Kenya (73.2%; *n* = 103; Nyambura Njuguna et al., 2017), Poland (62%; *n* = 81; Wierzbowska et al., 2020) and Malaysia (57.9%–89.3%); *n* = 28–152; Ngui et al., 2014; Tun et al., 2015). Such discrepancy in prevalence rates might be attributed to various factors like rural inhabitation, outdoor access, tropical to subtropical climatic conditions, poor feeding practices and unregulated deworming (Chalkowski et al., 2019; Genchi et al., 2021; Mircean et al., 2010).

In the current study, the prevalence of helminth parasites was higher than that of protozoa. This finding is per the outcomes from Italy (Mircean et al., 2010) and Malaysia (Ngui et al., 2014). *Ancylostoma* spp. were the most dominant parasites, similar to previous reports (Lima et al., 2017; Ramos et al., 2013; Wyrosdick et al., 2017). Notably, *A. tubaeforme* dominated *A. braziliensis*. Since current study has tropical to sub‐tropical climatic conditions, it provides optimal temperature and moisture necessary for egg hatching and larval development of the ancylostomatid parasite in the soil (Robertson & Thompson, 2002). In addition, most of the current felines have easy outdoor access. Thus, these two factors might have cumulatively increased the greater acquisition success of ancylostomatidae worm resulting in their higher prevalence. Similar to it, the current geo‐climatic condition also favours the quick development of unsegmented ova of ascarid into infective form in the soil. Interestingly, the prevalence of *T. cati* was higher than *T. leonina*. This discrepancy might have occurred due to the variance in their transmission route. Apart from ingestion of embryonated eggs and tissue of paratenic hosts (e.g., rodents, earthworms, cockroach and birds) by cats, newly born kitten acquire *T. cati* via pre‐ and perinatal route, but *T. leonina* does not follow this route and rarely occurs in kittens (Parsons, 1987). Epidemiologically, the shedding of a large number of eggs in each defecation contributes to the dissemination of the infection to other similar hosts and humans in the surrounding periphery, indicating that a large number of infected cats nearby human habitation can be a matter of public health concern. Furthermore, cats are also known to harbour several strongylid species like *Aelurostrongylus abstrusus*, *A. chabaudi*, *Angiostrongylus vasorum*, *Troglostrongylus brevior*, *T. subcrenatus* (Brianti et al., 2012; Giannelli et al., 2017; Gueldner et al., 2019; Jefferies et al., 2010; Mircean et al., 2010; Varcasia et al., 2014), and *Strongyloides* spp. like *Strongyloides felis*, *S*. *planiceps*, *S. stercoralis* and *S. tumefaciens* (Wulcan et al., 2019). However, we could only document three morphotypes of strongylid eggs and two morphotypes of *Strongyloides* eggs. Therefore, a detailed molecular study of these nematodes should be conducted in the future.

Considering cestodes, besides the common taeniid, the presence of *Dipylidium caninum*, *Hymenolepis* sp. and Archiacanthocephala had been the unique findings. Even though infection of *D. caninum* is common in pets like dogs and cats, their eggs are rarely released in the faeces although copromicroscopic technique is not reliable in detecting this cestode (https://www.cdc.gov/parasites/dipylidium/faqs.html). Acquisition of this worm could have occurred via the accidental ingestion of infected intermediate hosts like fleas (*Ctenocephalides felis*, *C. canis, Pulex irritans*) and lice (*Trichodectes canis*) by the current felines (Rousseau et al., 2022). In the same way, the hunting behaviour and preference of rodents, terrestrial insects and myriapods might have contributed to the acquisition of *Hymenolepis* sp. and Archiacanthocephala (Morand et al., 2007).

Regarding the protozoa, *Cystoisospora* spp. (*Cystoisospora felis* and *C. rivolta*) were the most dominant GI parasites. Since kittens are immunologically weak, they are highly susceptible to cocciodiosis (Dubey, 2018). An infected kitten can be contagious to other members of its group, and their contact with the mother's faeces may be a risk. Surprisingly, the presence of other coccidia like *Cryptosporidium*, *T. gondii/H. hammondi* and *Sarcocystis* spp. might be critical because these coccidia possess zoonotic potentiality, especially in the immunocompromised host (Shukla et al., 2006; Torda, 2001). Regarding it, a case of congenital toxoplasmosis had also been reported in Nepal in a 53‐day‐old baby who died within 6 days after the diagnosis and beginning of treatment (Rai et al., 2011) Similarly, four species of *Cryptosporidium* spp. like *Cryptosporidium felis*, *C. parvum, C. muris* and *Cryptosporidium* rat genotypes III and IV (Li et al., 2019; Taghipour et al., 2021) have already been identified from domestic cats. Furthermore, felines, being a predator, consumption of meat of infected animals put them at risk for *Sarcocystosis*. Although domestic felines can harbour *Sarcocystosis hirsuta, S. arieticanis, S. gigantea, S. fayeri, S. tenella, S. porcifelis* and *S. muris* and others (Bowman, 2014; Dubey, 1976; Soulsby, 2012), we could record four different morphotypes. This indicates an urgent need for further study that clarifies the taxonomic position of these coccidia in Nepalese cats.

Regarding Sarcodina, the presence of *Entamoeba* sp. (15%) has been distinctly noted although amoebiasis is usually uncommon in cats (Josephine, 1958). Epidemiologically, amoeba could be higher in those hosts that are rural inhabitants with poor lifestyles and feeding practices (Nath et al., 2015), which might be justifiable for their presence in the current felines.

Regarding sex‐wise bias, our report of higher prevalence in male cats contrasts, with those from Europe (Giannelli et al., 2017), Italy (Mircean et al., 2010) and Korea (Lee et al., 2019). The female cats usually remain within their home ranges, while the adult males are the free roamers. On attaining sexual maturity, male cats typically leave their home and become feral (Bikana Chaudhary personal communication, November 8, 2020). Besides, felines have promiscuous mating systems, that is, a single male mates with several females (Natoli et al., 2000). Thus, owing to the intimacy or proximity and higher contact rates with multiple sex partners, the possibility of the acquisition/transmission of parasites by male felines cannot be ignored (Altizer et al., 2003). Importantly, the testosterone hormone acts as an immune suppressor (Salvador et al., 1996), resulting in the susceptibility to the parasitic infection, although this should be checked further.

Further, similar to the findings from Canada (Joffe et al., 2011), Korea (Lee et al., 2019) and Brazil (Ramos et al., 2013), adult cats had a higher parasitic prevalence than the young ones, although conflicting results were obtained (Mircean et al., 2010; Nagamori et al., 2018). Adult cats usually roam in and around their home ranges (door‐to‐door, agricultural fields, roads and animal sheds) for food or mates, while the young usually remain within their home ranges. In addition to household feed, free‐roaming pets prefer hunting, and their diet includes mice, rats, insects, molluscs, small reptiles like the lizard, poultry, migrant birds, frogs and small mammals (Bonnaud et al., 2007; Gregory & Munday, 1976; Lanszki et al., 2016), which act as a reservoir or intermediate or paratenic hosts of the parasites (Anderson, 2000; Elmore et al., 2010; Singla et al., 2008). In contrast, young cats are provided with intense care by their mothers during their suckling and weaning periods that start from birth and continue up to the eighth week (Martin, 1986). Also, owners comparatively provide love and care to the young ones more than the self‐dependent adults. These factors could be the reason for the low prevalence rate and low diversity of GI parasites in young cats. Besides age and sex, the current study identifies outdoor lifestyle, and no or unknown history of medication and hunting behaviour of the felines contribute to greater parasitic acquisition.

Finally, 87.9% of the current cat populations had concomitant parasites, and a few of these had up to six parasites. This type of dominant polyparasitism is an interesting phenomenon in cats. For example, the presence of *Cryptosporidium* and *Tritrichomonas foetus* was associated with the severity of diarrhoea in cats (Gookin et al., 2001). Likewise, coinfection with *A. abstrusus* and *Eucoleus aerophilus* resulted in the death of a young Romanian cat (Giannelli et al., 2017). However, a feral cat infected with multiple parasite species had lesser *Toxocara* spp. load (Serrano & Millán, 2014) suggesting the probable negative or positive effects of polyparasitism in the hosts (Hoarau et al., 2020). The detection of the majority of *Cystoisospora* spp. and *Cryptosporidium* sp. in diarrhoeal stool and *A. tubaeforme* in diarrhoeal, mushy and lumpy, mixed, formed, and hard‐constipated stool indicates that these GI parasitism results in different pathologies in the hosts. Because the current study lacks histopathologic assays, further studies should confirm the causal association of coinfection in the host survival.

The current study is subjected to mainly two limitations, which should be considered. First, assessment of only a single faecal sample from a single feline and employment of smear assessment alone for parasitic determination might not be enough to accommodate the pre‐patent infections and the intermittent shedding of the parasitic cyst/eggs/oocysts/larva. It may underrate the quantification of prevalence as well as the concomitance of the parasites. A molecular study would support and elaborate the species‐level identification of the parasites. Second, the cross‐sectional method and use of a small and unbalanced sample size (*n* = 107) of domestic and feral cats may create difficulty in discussing the reason behind the discrepancy. However, we have strictly secured the quality control during field surveys and laboratory techniques, which we believe is the first copromicroscopic study in Nepal to address the unique GI parasites in domestic and feral cats.

## 5 CONCLUSION AND RECOMMENDATIONS

In conclusion, cats in the study area harbour a high prevalence of diverse GI parasites that vary according to age and sex. They are also associated with the predictors like outdoor lifestyle, absence of medication and hunting behaviour. The presence of major zoonotic parasites like *Ancylostoma*, *Cryptosporidium, Sarcocystis, Toxocara, Toxoplasma* and *Strongyloides* in the current cat populations indicates that the local people might be at risk of public health concerns. Therefore, an awareness programme for the local people and cat owners should be conducted for bringing up the treatment interventions, healthy feeding practices and proper disposal of cat faeces. Further, a One Health approach accompanied by molecular studies of faecal samples of cats, humans and domestic animals should be conducted to clarify the existence of the zoonotic epidemiology of the GI parasites.

## CONFLICT OF INTEREST

The authors declared no potential conflicts of interest with respect to the research, authorship and/or publication of this article.

## AUTHOR CONTRIBUTIONS

*Conceptualisation, formal analysis, investigation, methodology, writing–original draft, writing–review and editing*: Roshan Babu Adhikari.

*Formal analysis, writing–review and editing*: Madhuri Adhikari Dhakal. *Methodology, software, writing–review and editing*: Purna Bahadur Ale. *Investigation, writing–review and editing*: Ganga Ram Regmi. *Conceptualisation, formal analysis, methodology, supervision, writing–review and editing*: Tirth Raj Ghimire. All authors approved the final version of the manuscript.

## FUNDING INFORMATION

The authors received no financial support for the research, authorship and/or publication of this article. Laboratory facilities were provided by the Nepal Academy of Science and Technology.

## ETHICS STATEMENT

The required permission for collecting the faecal samples was issued by Ratnanagar Municipality and Ratnanagar Municipality Veterinary Service (Chitwan, Nepal; Permission No. 498/2077‐78). The study had been conducted using the stool samples defecated at the surface, and no experimental infection in cats was established during the work.

## Supporting information

Supporting Information

## Data Availability

The data that supports the findings of this study are available in the supplementary material of this article.
