# Supplementary material for: Survey on the prevalence of intestinal parasites in domestic cats (Felis catus Linnaeus, 1758) in central Nepal
Source: Vet Med Sci. 2022 Nov 8;9(2):559–71. doi: 10.1002/vms3.999 (PMC10029910; doi:10.1002/vms3.999)
Supplement: Supplementary file 1 — Supporting Information [file VMS3-9-559-s001.docx]

**Supplementary 1** Images of household and feral cats. A. Feral cat. B. Domestic cats. C. Domestic kitten.


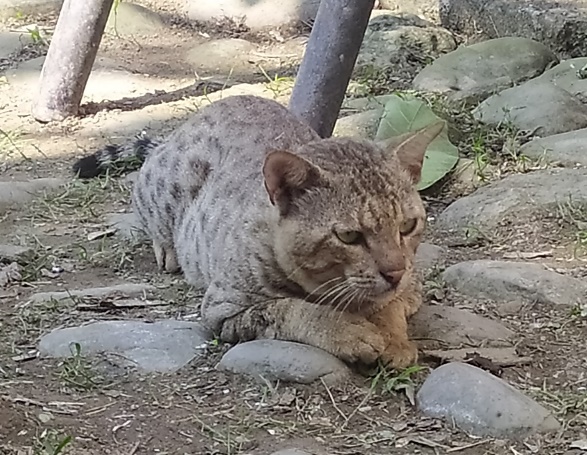


A


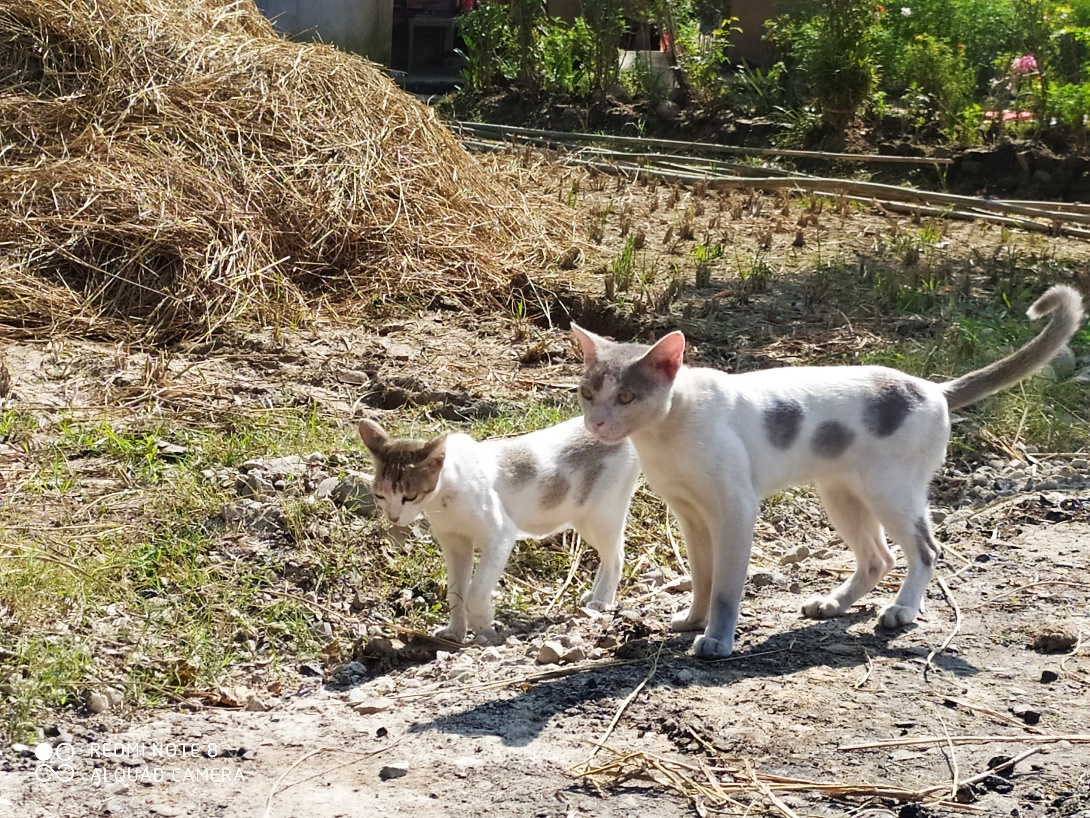


B


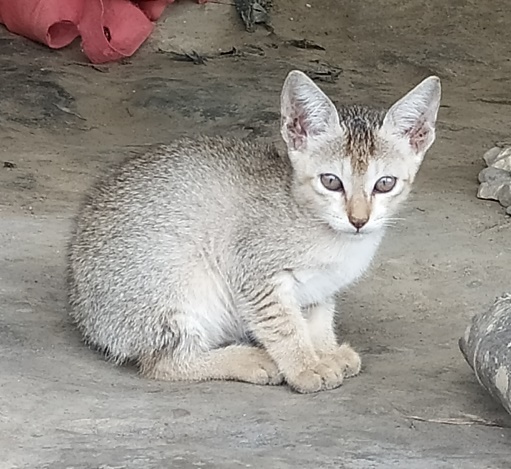


C

**Supplementary 2 Fecal consistency in household and feral cats. A:** Formed stool, **B:** Mixed stool, **C:** Hard and constipated stool, **D:** Mushy and lumpy stool, and **E:** Diarrheal stool.

| 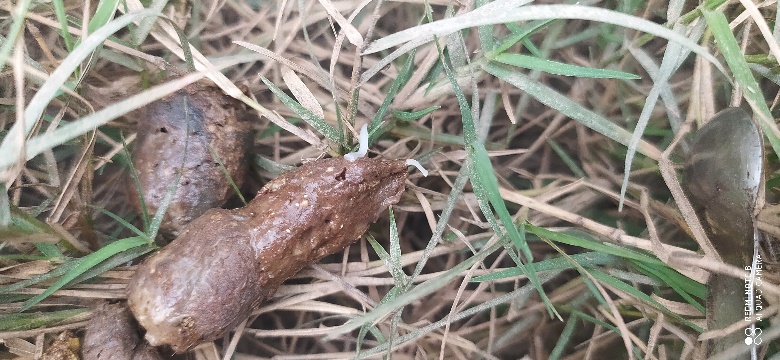 | 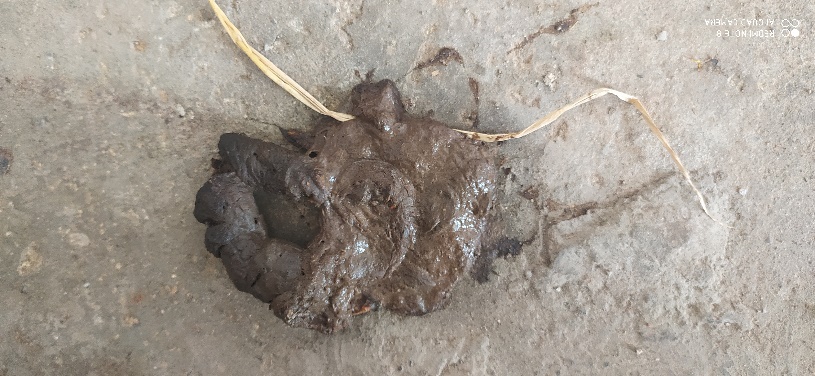 | 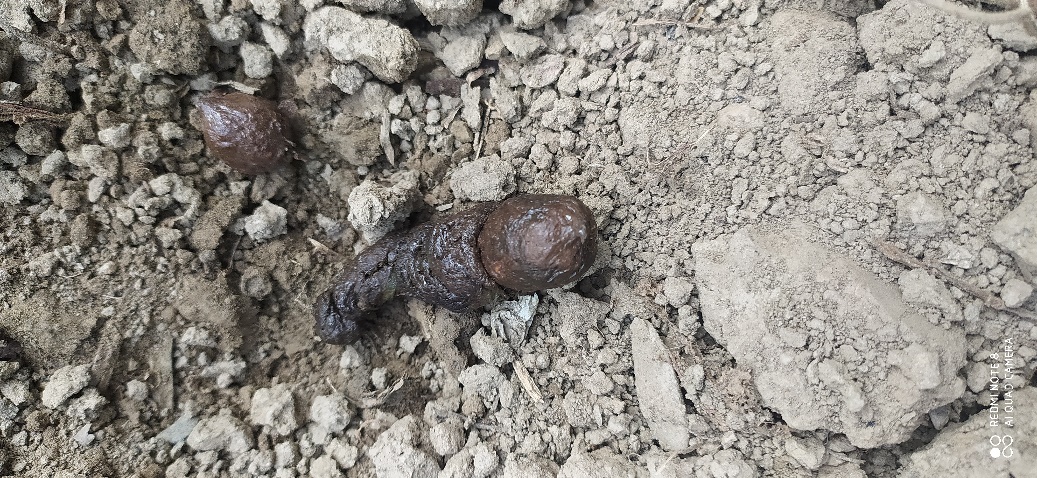 |
| --- | --- | --- |
| **A** | **B** | **C** |
| 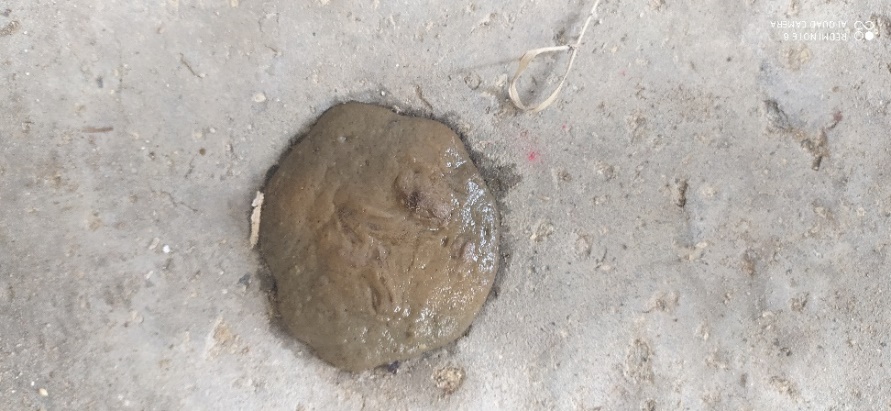 | 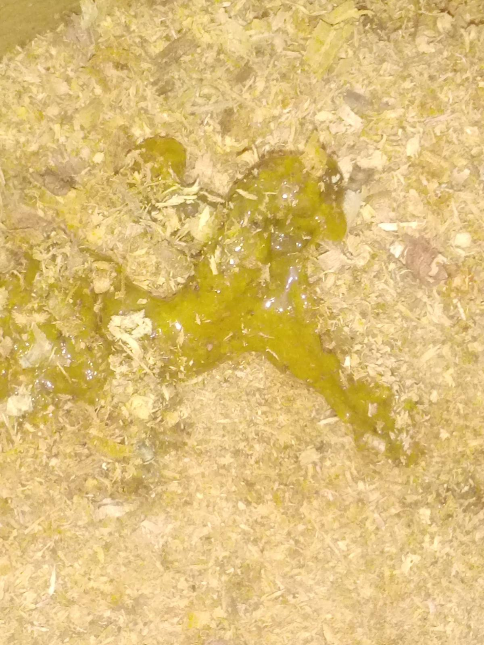 |  |
| **D** | **E** |  |

**Supplementary 3** Consistency of stool, its number, percentage, parasite-positive cases, and prevalence of GI parasites.

| **Consistency** | **Sample number (%)** | **Parasite positive** | **Prevalence** |
| --- | --- | --- | --- |
| Formed (normal) | 46 (43%) | 41 | 89.1% |
| Mixed | 33 (30.8%) | 33 | 100% |
| Mushy and lumpy | 16 (15%) | 16 | 100% |
| Hard constipated | 7 (6.5%) | 7 | 100% |
| Diarrheal | 5 (4.7%) | 5 | 100% |

**Supplementary 4** Prevalence of GI parasites among household and feral cats

|  | **Positi­­­­­­­­­­­­­­­­­­­­­­­ve and Prevalence rate** | | |
| --- | --- | --- | --- |
|  | **Household cats (n1=90)** | **Feral cats (n2=17)** | **Overall (N = 107)** |
| **Protozoa** | | | |
| *Cystoisospora rivolta* | 15 (16.7%) | 4 (23.5%) | 19 (17.8%) |
| *Cystoisospora felis* | 16 (17.8%) | 3 (17.6%) | 19 (17.8%) |
| *Sarcocystis* spp. | 11 (12.2%) | 6 (35.3%) | 17 (15.9%) |
| *Entamoeba* sp. | 12 (13.3%) | 4 (23.5%) | 16 (15%) |
| *T. gondii/H. hammondi* | 10 (11.1%) | 3 (17.6%) | 13 (12.1%) |
| *Cryptosporidium* sp. | 9 (10%) | 3 (17.6%) | 12 (11.2%) |
| Unknown coccidian | 0 (0)%) | 1 (5.9%) | 1 (0.9%) |
| **Helminths** | | | |
| *Ancylostoma tubaeforme* | 56 (62.2%) | 9 (52.9%) | 65 (60.7%) |
| *Toxocara cati* | 36 (40%) | 7 (41.2%) | 43 (40.2%) |
| *Ancylostoma braziliense* | 21 (23.3%) | 6 (35.3%) | 27 (25.2%) |
| Taeniid | 19 (21.1%) | 4 (23.5%) | 23 (21.5%) |
| *Capillaria* sp. | 11 (12.2%) | 3 (17.6%) | 14 (13.1%) |
| Strongyle | 8 (8.9%) | 3 (17.6%) | 11 (10.3%) |
| *Toxoascaris leonina* | 0 (0%) | 3 (17.6%) | 3 (2.8%) |
| *Dipylidium caninum* | 2 (2.2%) | 1 (5.9%) | 3 (2.8%) |
| *Hymenolepis* sp. | 2 (2.2%) | 0 (0%) | 2 (1.9%) |
| Archiacanthocephala | 2 (2.2%) | 0 (0%) | 2 (1.9%) |

**Supplementary 5** Prevalence of GI parasites associated with fecal consistency.

| **GI Parasites** | **Consistency (number)** | | | | | **Overall prevalence (%)** | **Chi-square tests (p<0.05)** |
| --- | --- | --- | --- | --- | --- | --- | --- |
|  | **Diarrheal (5)** | **Mushy and lumpy (16)** | **Mixed (33)** | **Formed (46)** | **Hard constipated (7)** |  |  |
| *Entamoeba* sp. | 0 (0%) | 1 (6.3%) | 6 (18.2%) | 9 (19.6%) | 0 (0%) | 16(15%) | - |
| *Cystoisospora rivolta* | 4 (80%) | 3 (18.8%) | 6 (18.2%) | 6 (13%) | 0 (0%) | 19 (17.8%) | - |
| *Cystoisospora felis* | 4 (80%) | 4 (25%) | 8 (24.2%) | 3 (6.5%) | 0 (0%) | 19 (17.8%) | - |
| *Sarcocystis* spp. | 1 (20%) | 4 (25%) | 6 (18.2%) | 6 (13%) | 0 (0%) | 17 (15.9%) | - |
| *Toxoplasma*/ *Hammondia* | 2 (40%) | 5 (31.3%) | 2 (6.1%) | 4 (8.7%) | 0 (0%) | 13 (12.1%) | - |
| *Cryptosporidium* sp. | 4 (80%) | 4 (25%) | 3 (9.1%) | 1 (2.2%) | 0 (0%) | 12 (11.2%) | - |
| Unknown coccidia | 0 (0%) | 0 (0%) | 1 (3%) | 0 (0%) | 0 (0%) | 1 (0.9%) | - |
| *Ancylostoma tubaeforme* | 3 (60%) | 7 (43.8%) | 20 (60.6%) | 30 (65.2%) | 5 (71.4%) | 65 (60.7%) | ns |
| *Toxocara cati* | 1 (20%) | 6 (37.5%) | 18 (54.5%) | 14 (30.4%) | 4 (57.1%) | 43 (40.2%) | ns |
| *Ancylostoma braziliense* | 2 (40%) | 6 (37.5%) | 9 (27.3%) | 9 (19.6%) | 1 (14.3%) | 27 (25.2%) | ns |
| Taeniid | 0 (0%) | 3 (18.8%) | 10 (30.3%) | 6 (13%) | 4 (57.4%) | 23 (21.5%) | - |
| *Dipylidium caninum* | 0 (0%) | 0 (0%) | 2 (6.1%) | 1 (2.2%) | 0 (0%) | 3 (2.8%) | - |
| *Capillaria* sp. | 2 (40%) | 2 (12.5%) | 7 (21.2%) | 3 (6.5%) | 0 (0%) | 14 (13.1%) | - |
| Strongyle | 0 (0%) | 2 (12.5%) | 4 (12.1%) | 5 (10.9%) | 0 (0%) | 11 (10.3%) | - |
| *Strongyloides* sp. | 0 (0%) | 1 (6.3%) | 3 (9.1%) | 3 (6.5%) | 0 (0%) | 7 (6.5%) | - |
| *Toxoascaris leonina* | 0 (0%) | 0 (0%) | 2 (6.1%) | 1 (2.2%) | 0 (0%) | 3 (2.8%) | - |
| Archiacanthocephala | 0 (0%) | 0 (0%) | 1 (3%) | 1 (2.2%) | 0 (0%) | 2 (1.9%) | - |
| *Hymenolepis* sp. | 0 (0%) | 0 (0%) | 0 (0%) | 2 (4.3%) | 0 (0%) | 2 (1.9%) | - |
